# A Case Study of Co-production Within a Mental Health Recovery College Dementia Course: Perspectives of A Person With Dementia, Their Family Supporter and Mental Health Staff

**DOI:** 10.3389/fresc.2022.920496

**Published:** 2022-06-22

**Authors:** Juniper West, Linda Birt, Danielle Wilson, Elspeth Mathie, Fiona Poland

**Affiliations:** ^1^Research Development Programme, Research and Development Department, Older People's Services, Norfolk and Suffolk NHS Foundation Trust, Norwich, United Kingdom; ^2^Faculty of Medicine and Health Sciences, School of Health Sciences, University of East Anglia, Norwich, United Kingdom; ^3^Centre for Research in Public Health and Community Care, University of Hertfordshire, Hatfield, United Kingdom

**Keywords:** dementia, recovery, co-production, peer support, mental health, service improvement

## Abstract

**Background:**

Undertaking co-production as a power-sharing way to improve mental health dementia services remains uncommon, suggesting opportunities to apply knowledge from lived experience of people with dementia, may often be missed. One barrier is stigma, assuming people with progressive cognitive impairment cannot manage this level of participation, support peers nor offer a “valid” perspective.

**Purpose:**

This paper shares knowledge gained from a service evaluation that explored various experiences of a person with dementia, their family supporter and mental health staff, involved in co-producing a course about “living well” with dementia, within a mental health Recovery College.

**Design:**

A qualitative, case study approach used semi-structured interviewing and inductive thematic analysis.

**Findings:**

Co-production activities generated a shared sense of positivity, pride and privilege, highlighting positive effects in breaking down the “them and us” barriers common in traditional healthcare professional-service user relationships. Each individual had both something to offer and something to gain during the process. Staff identified challenges in the co-production process; in that balancing all the voices during meetings could be complex at times, and the process overall required considerable time commitment.

**Conclusion:**

Taking part in co-production at an appropriate level and with peer support is a relational activity seen to be valuable in powerfully, yet gently, challenging stigma and assumptions around dementia. Findings show that while the process of co-production requires time and dedication, there is overall value in involving people living with dementia both in co-production and in peer support. This provided a straightforward and beneficial means to inclusively improve post-diagnosis support and care quality within a memory service.

## Introduction

The UK National Dementia Strategy (1) includes the objective of facilitating people with dementia and their families to engage in structured peer support and learning networks, enabling their active participation in improving local health services. In mental health dementia service improvement, facilitating people to “participate actively” means finding practical ways to draw on and value views from lived experience of people with dementia, and their family supporters. This lived knowledge is now used to inform individual care planning and to directly influence service or organisational level improvements (2). This element of a person-centred approach to dementia care (3) is frequently overlooked in everyday practice. Generally, involving people with dementia consists of consultation. This can be “tokenistic” if contributing does not change anything (4), it happens as a “one off” when a service is being re-configured (5) or it merely measures “patient experience” (6). In contrast, co-production is defined as an optimum level of engagement meaning people who use and people who work in public services share power to improve quality of services (4). For people living with dementia, actively participating in co-production (4) with appropriate support, to improve services, can help challenge negative stereotypes about dementia while improving care outcomes (7). The term “co-production” applied in its fullest sense in mental health, refers to true partnership working where staff and people with lived experience share power to plan, design and lead service improvements from the outset (8). The Dementia Engagement and Empowerment Project described positive examples of co-production, where groups of people living with dementia lead local and national policy and campaigns to reduce stigmatising language around dementia (9). Nonetheless, involving people with dementia specifically at any level of co-production, remains relatively uncommon, both in practices related to quality improvements of mental health services, and in published literature.

One area where co-production is flourishing, is in working-age adult mental health services. A recovery-focused, peer-supported approach is widely adopted to extend mental health care delivery outcomes beyond a narrow focus on symptom reduction, to help people rebuild meaningful, satisfying lives while living with the challenges caused by mental health difficulties (10). Five key concepts underpin the term “process of recovery” known as the CHIME framework (11). CHIME stands for **connecting** with others, **inspiring hope**, **maintaining** a positive **identity**, **finding meaning** in life beyond symptoms and **empowering control** over life and focusing on strengths (11). “Recovery Colleges” are adult educational settings for people who access mental health services, their families and staff. The Recovery College model offers co-produced courses to increase attendees' knowledge, skills and resilience in self-management of their own mental health. The CHIME framework and principles of co-production and peer support are in all aspects of Recovery College course design and delivery (10).

There is resonance between the CHIME framework and the value base of person-centred dementia care (7, 12–15). Person-centred care is defined as “valuing people with dementia and those who care for them” (3). To be person-centred, care should be individualised and understandings and actions should be derived from the perspectives of people with dementia. This may create more positive social-psychological environments (3). A UK scoping survey found that mental health organisations are incorporating dementia into Recovery College programmes (16). However, evidence from experiences of co-production with people with dementia in such programmes is scarce; only two published reports were found. Duff (17) reports on processes and possible benefits of using recovery principles in Recovery Colleges in Lincolnshire, suggesting a recovery approach could help reduce demand on services and help to raise quality of life for people with dementia. Cheffey et al. (18) (p 24) describe co-producing a dementia course for the Devon Recovery Learning Community, concluding that “*recovery orientated practice and co-production can and does work in the context of dementia.”*

A barrier to routinely embedding co-production, and peer support, within mental health dementia services, is overcoming stigma. Here the stigmatising assumption is that people with progressive cognitive impairment cannot manage this level of participation, nor provide a “valid” perspective. Participation activities such as re-designing services, recruiting staff or educating peers and clinicians (19) largely exclude people living with dementia. Yet evidence indicates that people with mild to moderate dementia continue to have significant self-awareness and agency in maintaining social connections (20).

Inclusively involving people with dementia in co-producing improvement planning has long been advocated as a person-oriented, empowering approach to health service delivery (1, 21). This innovative method is achievable if given time and care and can lead to improved dementia care practice (22, 23). It offers a way to build trust and understanding between service users and providers through enabling transparency and shared ownership of projects and services (24). People with dementia who have been involved in co-production report personal benefits such as increased self-confidence and self-esteem derived from having something to contribute (2). Similar benefits are evidenced throughout the field of patient and public involvement in dementia research (25, 26), where people with dementia are actively involved at all levels of participation, including as co-researchers (27–30).

This paper presents a descriptive single case study (31) using qualitative interview data from four individuals, all participants in a service evaluation: a person with dementia and their family supporter and two specialist dementia care staff, and reports on interviewees' experiences of both co-designing and co-delivering newly-formed Recovery College dementia courses. “Co-producing” entails both co-designing and co-delivering courses.

## Methods

### The Recovery College Dementia Course

Initially, three of the interviewees together with other peers and colleagues (“the group”) attended a dementia course running in another area of the Trust. Interviewees described this shared experience as supporting their own co-design plans. The group progressed to co-production work through meeting face-to-face eight times for 1.5 to 2 h, over a year, to shape, reflect on and co-develop learning aims, content and session practicalities within the context of their lived and learned experiences. They presented the course to the Recovery College's quality panel then went on to co-deliver courses. After the preliminary courses, the group shared reflections and made amendments before the next course. A complete course comprised 3 days over 3 consecutive weeks, and each day refreshed information from the preceding day to aid peoples' orientation and group cohesion. Course content included hearing directly from peer tutor's lived experiences and discussing topics such as: recovery using the CHIME framework (11), impact of dementia on relationships, stress management, and diagnostic disclosure. These aimed to facilitate group discussions. Following the onset of the COVID-19 pandemic, co-production meetings to amend and pilot course delivery took place through an online format using the Zoom videoconferencing platform (32). The courses mainly aimed to help attendees share experiences and learn about living well with dementia, including through peer support, also aiming to improve quality in their memory service.

### Design

The bounded nature of the sample, the important principle of hearing distinctly the experiences of people living with dementia, family supporters and staff supported taking a qualitative case study approach. Case studies can help understand different perspectives in context; here co-production of Recovery College courses with people living with dementia (33). The study described in this paper shares learning within a service evaluation to explore experiences and identify barriers and facilitators to involving people with dementia in service improvement in one mental health trust. The case was purposefully selected as the only group in the Trust involved in service improvement projects who were specifically co-producing their work. Approval was granted according to Trust research policy (NSFT/2019MH25-SE).

### Recruitment and Sample

Invitations and detailed information were sent to all staff facilitating the selected project. Two staff out of a possible four were available within the project timeframe and agreed to participate. Staff gained permission for [lead author] to follow-up with the person with dementia and their family co-producing the current course, to arrange interviews. Written informed consent, including for publishing findings, was obtained from all participants.

### Data Collection

Semi-structured interviews (*n* = 4) were conducted using topic guides developed by the authors. Strategies for conducting in-depth interviews with people with dementia were used (34) to ensure a guided yet conversational process (35). Interviews lasted approximately 40 min and were audio-recorded. Interviews with the person with dementia and their family supporter took place separately. There is evidence differences can occur in opinion between dyads (2, 36) therefore separate interviews placed emphasis on each person's needs and viewpoints in their own right, as opposed to the family supporter speaking only on behalf of the person with dementia (37).

### Data Analysis

Interviews were transcribed verbatim by the third author and a colleague within the Trust (TR). Participant identifiers were removed and the transcriptions reviewed for accuracy against the audio recordings by the lead author aiding familiarisation with data. Thematic analysis was used to inductively explore data for meaning (38) enabling us to generate subthemes (*n* = 36) and key themes (*n* = 11) from interviewees' words. Our constructivist approach ensured we distinguished organisational, public and personal “statuses” of participants alongside authors, person with dementia, family supporter and health professionals. This reflexive process alerted us to making assumptions or prioritising “professional points of interest” when interpreting participants' talk. This continuous reflexive process limited potential bias throughout analysis (38). Subthemes and themes emerging from processes and activities under study were therefore constantly revisited and reconstructed drawing on notes on findings from reflective discussions with co-authors from diverse disciplines, supporting trustworthiness of results.

## Results

Three overall areas for analysis were identified by organising the themes and subthemes from all interview data (Table 1) and identifying common areas. These were: **experiencing co-production as positive**; **creating a powerful experienced-based learning environment**; and **helping confront dementia together**. Interviewee experiences of co-producing the course are presented for each area.

**Table 1:** Key themes and subthemes.

**Participant/s**	**Key themes**	**Subthemes**
Person with dementia (*n* = 1)	Helping and being helped	[parent] not being helped didn't even talk about it help people deal with it
	Dementia out in the open	easier telling people it's just normal don't want people watching me
	Privileged and appreciative	with people that know all about it feeling good, comfortable, better talking about it all together
	Being with peers	there for what I am there for like being there comparing own with others similar position
Family supporter (*n* = 1)	Powerful learning this together	we needed to be with others sharing insights this works
	Pleased and proud	listened to and involved helping and being helped
	Unpicking all our attitudes	getting the message across can't cure it, live well with it getting the right words
	Comfortable and inclusive	time to feel at ease all naturally wary everyone started talking
Staff (*n*= 2)	Rewarding	kept me going expertise being valued wellbeing at work exciting work interest, enthusiasm, commitment
	Powerful learning	direct from lived experience uplifting enjoyed the focus more hopeful message
	Challenging, empowering person with dementia	being mindful balancing all the voices tricky feels collaborative
Total (*n* = 4)	(*n* = 11)	(*n* = 36)

### Experiencing Co-production as Positive

All four interviewees positively and explicitly valued their shared experience of co-producing learning materials, and their appreciation of each other's experiences. They described developing relationships during planning meetings and course days as being truly collaborative. *Experiencing co-production as positive* related to each person's motivations, sharing a sense of feeling good and being uplifted in the shift from clinical, to co-production relationships. The staff felt motivated by seeing co-production as central to their clinical roles, giving them time to re-centre themselves and their clinical teams on people and the lived experience of dementia. Staff described this way of working using words such as *exciting, interesting, valuing* and *rewarding*.

Staff participant (SP)1: *It's sometimes been the bit of the job that's kept me going…this is really powerful… it's really positive, it's a positive experience*.
SP2: *It's been really nice to work alongside in that more partnership way, with someone with dementia and a carer. And I think that just implicitly brings that message home about people are still people, and we all need that don't we? And to be reminded of that*.

The person with dementia talked about valuing this shift in relationships as they appreciated being involved, and the help this offered them personally. Talking about the meetings attended, to co-produce learning materials and delivering the course to peers, they used words such as *good, comfortable* and *a privilege*.

Interviewer (I): *What was your experience of being asked to be involved in the first place?*
Person with dementia (P): *I was so pleased. It really made me feel as if I was going to be able to take advantage of whatever was in front of me and I really do appreciate it*.
(I): *In what way?*
(P): *Because of what I saw with my [parent]. It was just behind everything. We didn't know to help [them] and I am getting all the help that is out there, and I feel so privileged*.

The family supporter, described being “pleased” and “proud” to be asked, and experiencing themselves as equal partners in decision-making as validating. Feeling actively listened to and involved was important in this process:

…* I was pleased that they asked me… I know they weren't listening and dismissing it because some of the things I suggested had been incorporated*.

That interviewees shared this sense of pride and privilege highlights positive impacts and how co-production can be effective in breaking down “them and us” barriers of traditional healthcare professional-patient relationships. The group could form positive and pragmatic working relationships where each individual had both something to offer and something to gain.

### Creating a Powerful Experienced-Based Learning Environment

Staff said they set up the co-production group in order to develop an inclusive learning environment which could help people's peers learn about living positively with dementia. The process of co-producing learning material then merged with co-delivering the course to their peers, and for each interviewee, being involved in both activities helped them individually. The staff and family supporter clearly expressed how powerful the experience was. While the person with dementia similarly conveyed this, they did so in less explicit terms. For example, the person with dementia mentioned being *amazed* by dementia being so openly talked about and contrasted this openness with their earlier experiences of social stigma with their parent. All interviewees specifically described their work together as creating a powerful learning environment, describing being enabled and being open to learning new things, having their ideas challenged by learning from one another. The person with dementia described feeling “good,” “comfortable,” and so “better” through sharing their lived experience “with people that know all about it.” However, they did not explicitly see themselves as a peer supporter, rather as someone being helped by openly talking about dementia. The family supporter could see this experience both helped their relative and benefitted themselves:

Family supporter: *I can't do it with [person with dementia - P] on my own. I couldn't have sat down with [P] and told [them] all that. [They] needed to be able to listen to other people saying the same thing, and I think it helped [them] with the professionals being there. As soon as [they] started talking to those that's when it made a difference*.

The staff drew similar conclusions as to the power of the process of co-production in the context of the learning environment created by and based on interviewees lived and learned experiences. For the co-production element, the power came from the interviewees involved. This was summed up by the family supporter who explained a sense of being able to give something back. Nonetheless, staff interviewees identified that it was tricky to balance the group's diverse needs and opinions. In particular, how to facilitate contributions and establish and maintain the presence and voice of the person with dementia throughout the process of co-producing the courses over time. These challenges were overcome through the staff being mindful and sharing the process, in co-producing small pieces of work, going away and in pairs, critiquing the work to bring back points for discussion and reflection. Solutions included setting the right pace, giving lots of time, space and support for the person with dementia to talk about their own, sometimes difficult, experiences of being diagnosed with, and living with dementia. All interviewees described co-production as a shared process of learning, reflecting, communicating, and challenging, rather than a one-off activity of question-asking. It required everyone to be open and inclusive and learn from each another and continuously reflect on their own thoughts and attitudes in a way that other forms of service user involvement might not.

### Helping Confront Dementia Together

The word “help” was used many times by the person with dementia, who alluded to feeling helpless in their earlier life when their parent experienced early-onset Alzheimer's dementia. That person talked about gaining personally through being with other people with dementia, within the context of the Recovery College course. Despite negative experiences of social stigma and fear of people behaving differently in response to their dementia-*I don't want people watching me*-being involved as an equal partner gave personal reassurance-*it's easier telling people, it's just normal*-and related a discovery that it would be *okay* to be open about personal lived experience of dementia and share this with other people.

Interviewer (I): *So you sharing your personal experience of living with dementia- having a diagnosis of dementia with other people who are on the course in the room, what's that been like, could you explain a bit?*
Person with dementia (P): *Until you have been with someone who was in the same position, like as if it wasn't, something that was hidden, something that was not talked about. It was coming out into the open and people like my [parent] were getting help rather than, ‘oh dear’. Because there was nothing. [My parent] wasn't on any medication, mind you, I am talking about years ago, … when [they] died, but yeah. [They were] my age actually. Yes and it sometimes comes back, you know, and I am thinking what a position I am in now, and the position [they were] in. So that's why I appreciate everything that I am being able to get hold of*.
I: s*o there is something there that you are saying about being in a place where there's other people who are going through the same?*
P: *Exactly. Exactly. The first [course], because there was someone there with dementia was amazing because it just wasn't talked about, now everybody is together and talking about it*.

This dialogue demonstrates how this course co-production process could provide a safe and accessible space for this person with dementia to raise the negative impact of experiencing social stigma with peers and care staff. For the staff, other motivating factors and service improvement ideas, were identified from their reflections from working with people during and post-diagnosis. Drawing on their clinical experience and ‘stepping into the shoes’ of the person going through the process of diagnosis, the staff spoke of memory service limitations such as its being exclusively professional-led. This co-production work enabled them to offer something else important, as well as a diagnosis:

Staff participant 1: …*there's only so much work you can do with somebody, with just you and them in a room, that needing something that will support them in being able to think about life with that diagnosis. And that, it just felt like has- that has to come from lived experience*.

Speaking about attendees on the course, the family supporter found it helpful to meet and hear directly from other people with dementia, to challenge their preconceived ideas that dementia was a fixed set of pre-determined features:

Family supporter: …*and the thing was, that they were both different, all three of them were different dementias which was helpful to start with, it made me realise right at the beginning of this, that dementia just wasn't affecting [their relative], it was affecting people in different ways…*

Realising this reassured the family supporter that not every person with dementia experiences every difficult symptom, encouraging them to use their learning experience to co-design the course to help others. The group were supported to reflect from their own perspectives on their own experiences of dementia-related helplessness. By coming together, they could face this helplessness and develop meaningful representations, often found helpful to acknowledge and share with others accessing their memory service.

## Discussion

This case study reports on the relatively novel experience of those involved in co-producing and co-delivering a Recovery College course for dementia. To-date there are only two published papers in this field (17, 18). These results add to this expanding but still innovative field of work, offering more developed insights, of connecting positively, experiencing hopeful messaging and facilitating openness, to characterise experiences of co-production facilitated in NHS dementia services drawing on an existing Recovery College structure.

### Connecting Positively

Framing a dementia course embedded in personal recovery principles implies accepting use of the positive term “recovery” in relation to dementia. The relevance of the CHIME framework (11) to living with dementia was much debated. The group members, through active listening and sharing of experiences and knowledge considered the challenges to the relevance of the recovery model and agreed how to present this to others. All interviewees described building shared appreciation for each other's lived and learned experience and presence through the co-production experience.

The findings resonate with the CHIME framework element “connecting with others” (11). Staff working in mental health have skills in balancing complex issues and needs, such as relationship dynamics, motivations, quality of interactions, language, and accessibility. Yet interpersonal processes involved in co-production relationships differ from those within clinical relationships. They require more skills in facilitating and managing co-production partners changing needs over time as dementia progresses. Importantly, this case study found that, despite actively co-producing work with people accessing memory services, the highly experienced staff interviewees, highlighted they needed to be reminded that people are still people. Bringing the topic of dementia into the Recovery College provided peer-led focused, reflective time to do this.

There remain significant barriers to co-production in dementia services becoming the norm. These ranged from clinicians' attitudes in developing skills to embed co-production in clinical practice, to finding time, alongside memory service waiting list pressures, to prioritise co-production. The staff interviewees described the process overall as demanding a considerable time commitment, and shift in the organisational culture to support the extra time, skills and resources clinical staff required (39). However, the family supporter described how the process was transformational in unpicking attitudes, so that co-production can bring about sustainable service-level change (40).

Recovery Colleges provide distinctive opportunities for people with lived experience of dementia to raise awareness of the impact of stigma and to share impactful lived experience with health professionals, to aid their professional reflective learning. The structure and processes involved supported genuine changes in power dynamics in participant relationships in practice, allowing staff to ask questions and seek advice from people with lived experience. This dynamic would not happen in routine clinical practice. This transformation highlights the potential for professional-service user relationships to be renegotiated, within Recovery Colleges and also the wider healthcare system. As Eley (5) (p 221) suggests, “*it is unfair and unrealistic to expect an individual to be the lone voice in a room of professionals, to tell their story coherently and to challenge the views of clinicians and other professionals with confidence.”* The numbers ratio of service users to staff appears essential to facilitate mutuality and connectedness in a co-production relationship. The inclusive design of Recovery Colleges addresses this.

### Experiencing More Hopeful Messaging About Living With Dementia

This case study indicated how co-creating a dementia learning environment can have a powerful and transformative effect on helplessness. Each interviewee conveyed from their own perspectives, feelings of helplessness in relation to acting with or on dementia, and also talked about how being involved in co-producing a course offered each person hope. For the person with dementia, hope came in demonstrably receiving help not historically offered to a relative with dementia. For the family supporter, hope came from meeting other people with lived experience and then being able to challenge their own preconceived ideas about dementia. The staff reported hope in potentially being able to address service gaps in user-led post-diagnostic support, expressing a strong message that the course helped people to process a diagnosis and this as being best (or only) done with guidance of people with lived experience. This indicates a need for a real, more hopeful message around dementia. This finding provides a challenge to known barriers to co-production with people living with dementia, namely staff “gatekeeping,” protecting people following a dementia diagnosis from perceived extra work or burden (41), and family supporters having the stronger voice (37).

### Facilitating Openness

Experiencing equality, both in acting within the co-production group, and also in the wider learning environment, which also included other people with dementia, was seen to help the person with dementia talk more openly about their own experience of living with dementia. For example, the person with dementia presented themselves as “just another person in the room” rather than “a peer supporter”. This experience of being accepted and of a safe environment allowed them to see and present dementia as part of their identity, but without it being their only identity and therefore to find meaning in life regardless of living with dementia. Having dementia was not seen here as a barrier to people meaningfully contributing to co-producing the course. This challenges the assumption that a person with progressive cognitive impairment would be inevitably “unable” to give a valid perspective. In the context of an equalising social-relational environment, the person with dementia was enabled to share knowledge through lived experience which was both empowering for the individual and valued by the other interviewees. Specifically, this included the person with dementia reflecting on how being involved in this work helped to safely bring dementia out in the open and to experience challenging long-entrenched stigma in a way that helped them personally to positively share their diagnosis with others. Overall, analytic themes were found to position co-production within this case as fundamentally a relational activity, which, in presenting and valuing lived experiences, can gently yet powerfully, challenge widely-held restrictive and negative assumptions about living with dementia.

### Limitations

Data were collected from only one group co-producing a course in a single organisation. We were, however, able to include four perspectives of the co-production relationship. Findings may reflect positive biases in that staff involved and the lead author, as a facilitator of person-centred dementia care learning, may have over-estimated the positive effects of recovery-focused practice and its role in helping people with dementia. The findings, however, are consistent with other reported experiences in the Recovery College dementia course field (17, 18). A further limitation was that only two staff out of a possible four were available to take part within the project timeframe, and that other people with dementia who were joining the group but not yet experiencing co-production could not be included. Finally, while criteria for Recovery Colleges are available (42), there is currently no universal model of co-production which may mean local interpretations of using and enacting co-production may vary across Recovery Colleges.

### Implications for Future Research

A more in-depth case study using multiple data collection methods could be used to ascertain the distinctive and relevant components in post-diagnostic support in dementia generated and supported through Recovery Colleges.

## Conclusion

Co-production with people with dementia, their family supporters and staff from mental health services remains a relatively novel activity. Few accounts in the literature illustrate co-production experiences of people with dementia, their family supporters, and staff working in mental health services. This case study details ways in which co-producing a dementia course can offer a positive experience for all involved. Using the CHIME framework (11) built staff confidence in facilitating this work, to apply and fulfil their practice values, showing how a recovery-focused approach can be relevant to people adjusting to a dementia diagnosis. The powerful, inclusive shared learning environment this group created enabled each interviewee to learn new things including from one another, by challenging each other's ideas. Valuing as knowledge, the experiences of a person living with dementia was seen to generate novel and distinctive learning materials, processes and outcomes. Confronting dementia challenges together in this way within a recovery principles context helped reflect on feelings of isolation linked to adjusting to a dementia diagnosis.

## Data Availability Statement

The raw data supporting the conclusions of this article will be made available by the authors, without undue reservation.

## Ethics Statement

Ethical review and approval was not required for the study on human participants in accordance with the local legislation and institutional requirements. The patients/participants provided their written informed consent to participate in this study.

## Author Contributions

The research topic was conceived by JW, supervision and additional research expertise provided by FP, EM, and LB. JW undertook the initial analysis and emerging results were refined in discussion with DW, FP, EM, and LB. The initial manuscript was prepared by JW and all authors provided critical comment and text editing support.

## Funding

This is a summary of research funded by the National Institute for Health Research (NIHR) Collaboration for Leadership in Applied Health Research and Care East of England (CLAHRC EoE) Programme, recommissioned as the NIHR Applied Research Collaboration (ARC) EoE from 1st October 2019. The views expressed are those of the author(s), and not necessarily those of the NHS, NIHR or Department of Health and Social Care.

## Conflict of Interest

The authors declare that the research was conducted in the absence of any commercial or financial relationships that could be construed as a potential conflict of interest.

## Publisher's Note

All claims expressed in this article are solely those of the authors and do not necessarily represent those of their affiliated organizations, or those of the publisher, the editors and the reviewers. Any product that may be evaluated in this article, or claim that may be made by its manufacturer, is not guaranteed or endorsed by the publisher.
